# Regularized logistic regression with network-based pairwise interaction for biomarker identification in breast cancer

**DOI:** 10.1186/s12859-016-0951-7

**Published:** 2016-02-27

**Authors:** Meng-Yun Wu, Xiao-Fei Zhang, Dao-Qing Dai, Le Ou-Yang, Yuan Zhu, Hong Yan

**Affiliations:** School of Statistics and Management, Shanghai University of Finance and Economics, Guoding Road, Shanghai, 200433 China; Key Laboratory of Mathematical Economics SUFE, Ministry of Education, Guoding Road, Shanghai, 200433 China; School of Mathematics and Statistics & Hubei Key Laboratory of Mathematical Sciences, Central China Normal University, Luoyu Road, Wuhan, 430079 China; Intelligent Data Center and Department of Mathematics, Sun Yat-Sen University, Xingang West Road, Guangzhou, 510275 China; College of Information Engineering, Shenzhen University, Nanhai Avenue, Shenzhen, 518060 China; School of Automation, China University of Geosciences, Lumo Road, Wuhan, 430074 China; Department of Electronic and Engineering, City University of Hong Kong, Tat Chee Avenue, Hong Kong, 999077 China

**Keywords:** Protein-protein interaction network, Edge-biomarker discovery, Network-based pairwise interaction, Node degree, Adaptive elastic net

## Abstract

**Backgroud:**

To facilitate advances in personalized medicine, it is important to detect predictive, stable and interpretable biomarkers related with different clinical characteristics. These clinical characteristics may be heterogeneous with respect to underlying interactions between genes. Usually, traditional methods just focus on detection of differentially expressed genes without taking the interactions between genes into account. Moreover, due to the typical low reproducibility of the selected biomarkers, it is difficult to give a clear biological interpretation for a specific disease. Therefore, it is necessary to design a robust biomarker identification method that can predict disease-associated interactions with high reproducibility.

**Results:**

In this article, we propose a regularized logistic regression model. Different from previous methods which focus on individual genes or modules, our model takes gene pairs, which are connected in a protein-protein interaction network, into account. A line graph is constructed to represent the adjacencies between pairwise interactions. Based on this line graph, we incorporate the degree information in the model via an adaptive elastic net, which makes our model less dependent on the expression data. Experimental results on six publicly available breast cancer datasets show that our method can not only achieve competitive performance in classification, but also retain great stability in variable selection. Therefore, our model is able to identify the diagnostic and prognostic biomarkers in a more robust way. Moreover, most of the biomarkers discovered by our model have been verified in biochemical or biomedical researches.

**Conclusions:**

The proposed method shows promise in the diagnosis of disease pathogenesis with different clinical characteristics. These advances lead to more accurate and stable biomarker discovery, which can monitor the functional changes that are perturbed by diseases. Based on these predictions, researchers may be able to provide suggestions for new therapeutic approaches.

**Electronic supplementary material:**

The online version of this article (doi:10.1186/s12859-016-0951-7) contains supplementary material, which is available to authorized users.

## Background

Biomarker discovery for cancer based on multiple molecular data, such as gene or protein expression data, has become a major strategy in biomedical fields for personalized medicine. Diagnostic and prognostic biomarkers have the potential to provide deeper insights into disease pathogenesis [1]. Revealing the mechanisms of disease initiation and progression can be valuable for the selection of new therapeutic approaches and the prediction of later clinical benefit [2, 3].

With the increasingly accumulated “omics” (e.g. genomics, transcriptomics and proteomics) data generated from high-throughput technologies, extensive variable selection methods such as lasso [4] and elastic net [5] have been proposed to select relevant biomarkers for disease diagnosis or prognosis, where the genes or proteins are regarded as variables. These methods often focus on the variables that can discriminate patients in the training set with the outcome measured by categorical variables, such as normal and disease, and are able to predict well unseen patients [6]. It is a computational and statistical challenge to detect reliable and useful biomarkers for diseases with the relative small sample size and high dimensionality of most molecular data [7]. With a wealth of publicly molecular data for the same disease, it is common that the biomarker signatures from different studies have few overlaps [8]. This is partly because that many different biomarkers have similar discriminatory power. Another reason is the instability of the variable selection method towards the used samples. The low reproducibility of these signatures often result in the difficulty of achieving clear biological interpretation. On the other hand, the criterion that identifying differentially expressed genes often neglects non-differentially expressed genes which play a central role in the molecular mechanism of complex biological phenomena by interacting with other genes [9]. Complex diseases are multifactorial biological events manifested through changes in expressions of many individual genes and proteins, and mediated through complex interaction mechanisms [10–12]. This connectivity implies that the impact of a specific genetic abnormality is not restricted to the activity of the gene product that carries it, but can spread along the interactions and alter the activity of the connected gene products [13]. Therefore, changes of these interactions of which the involved genes or proteins are not differentially expressed may also result in the different states of a biological system.

In this situation, traditional variable selection methods (e.g. lasso and elastic net) which are based on the additive model are insufficient for predicting an outcome of interest. To overcome this limitation, a regression model with pairwise interactions between those variables is proposed by Bien et al. to select a subset of variables and interactions between variables that is predictive of the response [14]. However, their work is based on an assumption that an interaction is allowed into the model only if at least one of the corresponding variables is also in the model. This restriction makes it impossible to identify the interactions with the non-differentially expressed variables. Zhang et al. propose a new idea based on a new vector representation of an edge to identify edge-biomarkers which are the differentially correlated molecular pairs with optimal classification abilities [9]. In their work, the correlation of each molecular pair is depicted by two new coupled variables, which makes the dimension of the new space increase to *p*(*p*−1), where *p* is the number of original variables. The computation is so time consuming that a preliminary variable screening is needed. In addition, the correlations used in these methods are only based on the expression data, which are not sufficient to reveal the physical interaction between two genes or proteins.

To deal with limitations in existing methods, a promising direction is to integrate expression profiles with rich biological knowledge in network datasets. A useful technique is to incorporate biological networks into variable selection process by a network-constrained regularization procedure, where the network is represented as a graph and its corresponding Laplacian matrix [15–17]. Another idea is to use these networks as Markov random field priors to guide the selection of relevant genes [18, 19]. The contribution of the network information used by these network-regularized approaches is to ensure smoothness of the coefficients on the network. They do not explicitly select the important interactions. Strategies to identify gene subnetwork or module have been proposed by integrating gene expression data and biological networks [3, 20–22]. Guo et al. treat the gene expressions within a functional module as an integrative data point based on the Gene Ontology (GO) enrichment analysis [22]. Some algorithms start from “seed” genes with highly differentially expressed in the network to generate the subnetwork-biomarkers, resulting in the neglect of the subnetworks with non-differentially expressed genes but different interactions [20, 21]. Moreover, Das et al. identify overlapping functional modules based on the topological properties of the protein interaction network by some clustering algorithms, and then use the elastic-net-based regression model to detect the differentially expressed functional modules [3]. The prognosis prediction results and the identified differentially modules may partly depend on the selected clustering algorithms. Another new perspective to reveal potential mechanisms altering the biological states is analyzing the comparison of biological networks across a set of conditions, and identifying the subnetwork-biomarkers which are differentially co-regulated [23, 24].

**Fig. 1:**
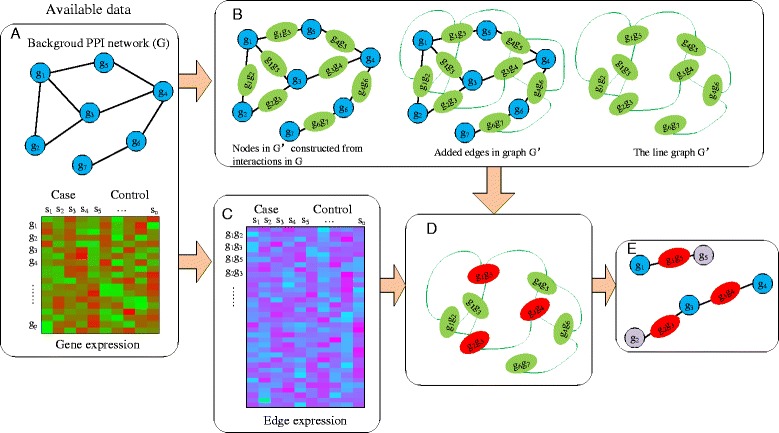
Identification of an edge biomarker using the regularized logistic regression with network-based pairwise interaction via adaptive elastic net. **a** The available data, including expression data and PPI network data. **b** A line graph *G* ^′^(*V* ^′^,*E* ^′^,*W* ^′^) is constructed based on the similarities *S*(*e*_*ik*_,*e*_*jk*_) which are treated as the weights for the new edges of graph, where the elements of the new node set *V* ^′^ are the interactions in the biological network. **c** The expression of each gene is mapped to the edge of the network in different conditions by integrating both the network and expression information. **d** If the expression of the interaction change in the different states of a biological system, the edge is labeled in red, while green if not. **e** The informative edges identified by our model. The genes with differential expression is marked in purple, while blue if with non-differential expression

Motivated by the challenges posed by the instability and complex interactions in high-dimensional gene expression datasets, this study proposes a regularized logistic regression with network-based pairwise interaction via adaptive elastic net for biomarker identification (Fig. 1). The model embeds variable selection in the classifier construction process with the advantage that the differentially interactions can be used directly to predict the states of the new samples. Instead of identifying discriminative genes or functional modules with differential expression, where the modules often need to be determined in advance by some algorithms such as in [3, 22], we focus on the detection of gene pairs which exhibit different positive or negative interactions, thus the performance of the proposed method will not depend on the module detection algorithms. The results based on experimental characterization of mutant alleles in various disorders show that the biochemical and physical interactions which are represented in models as edges are correlated with distinct structural properties of disease proteins and disease mechanisms [25, 26]. The model only considers the interactions belonging to a protein-protein interaction (PPI) network. The interactions based on both high-dimensional “omics” data and biological network can help to filter out the correlations between expression that have no underlying biological causality, which make the model have a low complexity [27]. The integrated information can lead to the improvement of both predictive accuracy and interpretability of the selection results [18]. In addition, different from the elastic-net-based regression model used by Das et al. in [3], our model includes an extension of the standard adaptive elastic net to consider the degree of proteins based on the line graph and the assumption that disease-genes tend to have high degrees in biological networks [28, 29]. This formulation leads to more stable biomarkers because the model is less reliant on the expression data. By applying the new model to six publicly available breast cancer datasets, we show that the algorithm is robust against the inclusion or exclusion of some patients on variable selection process at both gene and functional levels. Furthermore, our method can achieve competitive classification results with the state-of-the-art algorithms on detecting different responses to a certain survival time. The relevance of many identified biomarkers with breast cancer have been verified through biochemical or biomedical research. The Gene Ontology (GO) analysis further indicate the significant biological and functional correlations of the edge-biomarkers.

## Methods

### Regularized logistic regression with network-based pairwise interaction

Suppose that there are *n* independent *p*-dimensional observations, with binary response vector *y*=(*y*_1_,⋯,*y*_*n*_)^*T*^ and design matrix *X*=(*x*_1_,⋯,*x*_*n*_)^*T*^, where *x*_*i*_=(*x*_*i*1_,*x*_*i*2_,…,*x*_*ip*_) and *y*_*i*_∈{0,1}. Let *p*(*x*_*i*_) represents the class-conditional probability for observation *i* when *y*_*i*_=1 at particular parameters *β*_0_,*β*=(*β*_1_,⋯,*β*_*p*_)^*T*^. In order to identify the biomarkers which have low discriminative power but play a central role in the molecular mechanism of complex biological phenomena by interacting with other genes, we define *p*(*x*_*i*_) through network-based pairwise interactions between variables as follows

(1) $$ p(x_{i})=Pr(y_{i}=1|x_{i})=\frac{1}{1+e^{-(\beta_{0}+\sum_{j\sim k} \beta_{jk} x_{ij}x_{ik})}},  $$

where $\sum _{j\sim k}$ denotes the sum over all unordered pairs {*j*,*k*} for which *j* and *k* are adjacent on the certain undirected biological network. The pairs {*j*,*k*} and {*k*,*j*} are regarded as the same pair and will be treated in the model only once. The proposed regularized logistic regression model maximizes the penalized log-likelihood

(2) $$ \frac{1}{n}\sum_{i=1}^{n}\left[y_{i} \log p(x_{i})+(1-y_{i})\log (1-p(x_{i}))\right]-\lambda P_{\alpha}(\beta),  $$

where *P*_*α*_(*β*) is a penalty function which can shrink some components of *β* to zero for some appropriately chosen *λ* and *α* [30]. Compared with the two-way interaction model in [14] which includes *p*+*p*(*p*−1)/2 variables, the proposed model only takes account into the pairwise interactions or edges in the biological network, whose number is much less than *p*(*p*−1)/2. This form not only makes the model have an advantage in complexity, but also combines biological network information with genomics or proteomics datasets. The integrated information can reduce the impact of the noise from gene expression data on the process of estimating the biomarkers. Thus, the model will be less sensitive to the samples used in the phase of gene selection.

There are many penalty functions which are suitable for the regularized logistic regression model, such as lasso [4], adaptive lasso [31], and elastic net [5]. Zou and Zhang proposed the following adaptive elastic net as an improved version of the elastic net for analyzing high-dimensional data using a combination of the *L*_2_ penalty and the adaptive *L*_1_ penalty,

(3) $$ P_{\alpha}(\beta)=\sum_{j=1}^{p}\left[\frac{1}{2}(1-\alpha){\beta_{j}^{2}}+\alpha w_{j} |\beta_{j}|\right],  $$

where $\{\omega _{j} \}_{j=1}^{p}$ are the adaptive data-driven weights [32]. The parameter *α* is typically fixed to select a trade-off between adaptive lasso penalization and ridge regression, while *λ* is varied to tune the model [33]. In order to overcome the problem that the correlated variables have different coefficients, the value of *α* should not be too close to one [34]. Existing studies show that this adaptive elastic net penalty not only have group effect which can select groups of correlated variables, but also can identify a number of representative biomarkers with clear biological meanings and achieve effective classification [32, 35, 36]. Instead of computing the weights by

(4) $$ w_{j}=\left(|\hat{\beta}_{j}^{EN}|\right)^{-r},  $$

where *r* is a positive constant and $\hat {\beta }_{j}^{EN}$ is the elastic net estimator, we calculate the weight for each gene according to the biological network information, resulting in the higher stability of the gene selection process. The specific method will be introduced in later section.

The intercept parameter *β*_0_ and regression coefficient vector *β* can be estimated by the maximizer of objective function (2). The objective function can be written in the form of a concave function of the parameters as follows

(5) $$ {{ \begin{aligned} {}\frac{1}{n}\sum_{i=1}^{n} \!\left[\!y_{i}\!\left(\beta_{0}\,+\,\sum_{m=1}^{M} \tilde{\beta}_{m} \tilde{x}_{im}\right)\!-\log\!\left(\!1\,+\,e^{\beta_{0}+\sum\limits_{m=1}^{M} \tilde{\beta}_{m} \tilde{x}_{im}}\!\right)\!\right]\,-\,\lambda P_{\alpha}(\beta), \end{aligned}}}  $$

where

(6) $$ \tilde{x}_{im}=x_{ij}x_{ik}  $$

for the edge *j*∼*k* of the network, and *M* is the number of the edges that is much less than *p*(*p*−1)/2. The Newton algorithm can be used to approximate the objective function (5) by a second-order Taylor series expansion at current estimates [30]. Then the maximization of (5) is equivalent to a penalized weighted least-squares problem which can be solved by an efficient coordinate descent algorithm. Based on this algorithm, the matlab code “glmnet” can provide a path of solutions for a decreasing sequence of values for *λ* given a fixed value of *α*.

### The weights for adaptive elastic net

For the regularized logistic regression with network-based pairwise interaction via adaptive elastic net (RLRNPI-AEN) (5), instead of computing the weights by Eq. (4), we calculate the weights based on the characteristics of networks topology.

Firstly, we present a biological network by a weighted graph *G*(*V*,*E*,*W*), where *V* is the set of *p* nodes that correspond to the *p* variables, *E*={*e*_*ij*_} is the set of edges between two nodes, and *W* is the set of weights of the edges. The edge weight can be used to measure uncertainly of the edge between vertices. For a network without explicit edge weights, the weight of every edge is set to one. A weighted adjacency matrix *A* can be used to represent the weighted edges, where *A*_*ij*_=*w*_*ij*_ if there exists a edge between nodes *i* and *j* and *w*_*ij*_ is the weight of the edge, and *A*_*ij*_=0 otherwise. For an undirected graph, the adjacency matrix *A* is symmetry. The degree of node *i* is defined as $d_{i}=\sum _{j=1}^{p} a_{\textit {ij}}$.

Secondly, we construct a line graph for the interactions (edges) which describes the relationships between overlapping edges, where each node may belong to more than one edge. The similarity between two edges is referred to the measure stated in [37]. The set that includes node *i* and its neighbors is denoted as *n*_+_(*i*). If two edges do not share a same node, their similarity will be set to zero. Otherwise, the similarity between edges *e*_*ik*_ and *e*_*jk*_ is defined as

(7) $$ S(e_{ik},e_{jk})=\frac{|n_{+}(i)\bigcap n_{+}(j)|}{|n_{+}(i)\bigcup n_{+}(j)|}.  $$

Since the shared node *k* provides no additional information, it does not present in the definition [37]. This definition is based on the assumption that edge pairs with a shared node are expected to be more similar than those unconnected pairs, and the similarity between two connected edges relies on the fraction of common neighbors that their unshared nodes have. Then, we can construct a line graph *G*^′^(*V*^′^,*E*^′^,*W*^′^), where *V*^′^ consists of nodes which correspond to the interactions in the biological network (i.e. *e*_*ij*_), and *E*^′^ consists of the edges in this line graph. There is an edge between *e*_*ik*_ and *e*_*jk*_ since they share a common node and the weight of this edge is *S*(*e*_*ik*_,*e*_*jk*_) (Fig. 1). Since an interaction (node in line graph *G*^′^) that includes high degree nodes (node in original graph *G*) will be more likely to have common nodes with other interactions, it will have high degree in the line graph *G*^′^. Studies have shown that disease-genes are always characterized by large degrees in biological networks, and are more likely to interact with other disease-genes [28, 29]. In addition, the variation of the high degree nodes will impose more influence on the action of the whole network, since they have more interacting partners. Therefore, we prefer to pick out the genes with higher degrees. The weights for adaptive elastic net (3) in objective function (5) are set to be inversely proportional to the degrees as follows,

(8) $$ w_{j}=\left(d'_{j}\right)^{-r}  $$

where $d^{\prime }_{j}$ is the degree of the interaction *j* in the line graph *G*^′^ and *r*≥0 is a parameter that controls the weights. This definition tends to select the interactions which include nodes with high degrees in the biological network. When *r*=0, the adaptive elastic net penalty turns back to the elastic net. The larger value of *r* will promote the stability of the gene selection process because of the less dependence of the model on the samples. However, a too large *r* will generate a model that only focuses on the degrees of genes and neglects their discriminating abilities. We will discuss the specific method for the determination of *r* in the next section. The main steps of the proposed method are summarized in Algorithm ??.



### Evaluation metrics

We use three metrics to assess the performance of various methods. Firstly, the classification performance of the model on the certain dataset is measured by the area under the ROC curve (AUC). Similar to [38] and [39], we perform ten times ten-fold cross-validation experiments for each dataset to minimize sampling noise. The repeated ten-fold cross-validation estimator is found to have better performance than the.632+ bootstrap estimator which suffers from a bias problem for large samples as well as for small samples [40]. Given a fixed *α* and *r*, another 10-fold validation is used for the selection of the parameter *λ* based on the training set (90 *%* of the original dataset), and the AUC is computed on the remaining testing set (10 *%* of the original dataset). Similar to that in [3], for each ten-fold cross-validation, the median AUC value is computed over the ten experiments.

Secondly, as mentioned in [41], the stability of gene selection process is compared on different samples in three settings: the soft-perturbation, the hard-perturbation, and the between-datasets settings. For the regularized logistic regression with network-based pairwise interaction, the set of selected interactions will be transferred to gene set, where the genes which belong to different interactions will be considered only once. The soft-perturbation setting evaluates stability with respect to small perturbation of the training set, where a pair of training sets are randomly generated with 80 *%* overlap. Through the 10-fold cross-validation for *λ*, the following Jaccard coefficient between the two gene sets *G*_1_ and *G*_2_ is considered as the index for the stability of gene selection process,

(9) $$ JC=\frac{|G_{1}\bigcap G_{2}|}{|G_{1}\bigcup G_{2}|}.  $$

The median is evaluated over the 20 repeats of the above random sampling. The hard-perturbation setting is referred to a procedure that randomly subsamples each dataset into pairs of subsets with no sample in common. A similar process is used to compute the median Jaccard coefficient. The between-datasets setting considers each dataset independently, using all samples on each dataset. In this setting, the genes are ranked by the number of times that the variables become selected when *λ* decreases. For each pair of the datasets, we compute the overlap of the two sets of the top *k* genes. The results are computed by taking the median stabilities of the total pairs of all the datasets.

Thirdly, the functional stability of a gene selection process is also assessed in the above three settings. We measure the functional similarity of two gene sets by the method stated in [42] which is based on the similarity of the GO terms of the genes. The three GO domains, biological process (BP), cellular component (CC) and molecular function (MF) are considered respectively.

### Datasets

Breast cancer remains the most prevalent cancer among women in many countries. All the six datasets used in this study are measured on Affmetrix HGU133 microarrays, and each dataset includes 22283 transcripts. They are available through the Gene Expression Omnibus (GEO) database. A summary of these datasets are listed in Table 1. Except datasets GSE1456, GSE6532, GSE4922 which are downloaded as ready normalized, we preprocess the other three datasets by background correcting, quantile normalizing and log2 transforming using R package “preprocessCore” [43] (Bolstad, B: Probe level quantile normalization of high density oligonucleotide array data, unpublished). The probesets which do not have corresponding gene names are not considered and removed from our datasets, and the expression values for probesets that map to the same gene are averaged, resulting in 12754 genes. Both survival time information and the corresponding event indicator are used to divide samples into two classes according to whether patients develop a reported metastasis/relaspe/disease event within 5 years or are free of metastasis/relaspe/disease at least 7 years. A high-quality protein-protein interaction (PPI) network in H. sapiens from the High-quality INTeractomes (HINT) database (version: 06/03/2013) is used for constructing the pairwise interaction and calculating the weights in adaptive elastic net [44]. We only consider genes both in expression data and interaction network. In this PPI network, there are 18864 pairwise interactions including 6342 genes after self loops are removed. All datasets used in this study are obtained from papers and databases that have been already published and required no ethics approval.

**Table 1:** Overview about employed breast datasets

Dataset	Publication	# patients	Classification	# patients of each class
GSE2034	[62]	242	time to relapse ≤5 y & relapse =True	95
			time to relapse >7 y & relapse =False	147
GSE1456	[63]	111	time to relapse ≤5 y & relapse =True	35
			time to relapse >7 y & relapse =False	76
GSE11121	[64]	125	t.dmfs ≤5 y & e.dmfs =True	28
			t.dmfs >7 y & e.dmfs =False	97
GSE6532	[65]	178	t.dmfs ≤5 y & e.dmfs =True	51
			t.dmfs >7 y & e.dmfs =False	127
GSE4922	[66]	204	DFS.time ≤5 y & DFS.status =True	70
			DFS time >7 y & DFS.status =False	134
GSE12093	[67]	79	DFS.time ≤5 y & DFS.status =True	12
			DFS.time >7 y & DFS.status =False	67

t.dmfs denotes the time for distant metastasis-free survival and e.dmfs is the corresponding event indicator. DFS.time denotes the time for disease-free survival and DFS.status is the corresponding event indicator

## Results and discussion

In the experiments, we apply seven algorithms, namely, regularized logistic regression via elastic net (RLR-EN) [30], regularized logistic regression via adaptive elastic net (RLR-AEN), regularized logistic regression with network-based pairwise interaction via elastic net (RLRNPI-EN), logistic regression with differentially expressed genes selected using the LIMMA package (limma) [45, 46], elastic-net-based prognosis prediction (ENCAPP) [3], SVM-based classification with average expression profile of pathways (SVM-AEP) [22] and RLRNPI-AEN on six human breast cancer datasets (The descriptions of RLR-EN and RLR-AEN are presented in Additional file 1). For limma, a 10-fold cross-validation is used to find the suitable number of differentially expressed genes. For ENCAPP, there are two parameters *α* and *λ* in the elastic net model, where *α* is fixed as described below and *λ* is chosen by a 10-fold cross-validation. SVM-AEP is implemented with R package “netClass” using default parameters [39].

### Parameter settings

**Fig. 2:**
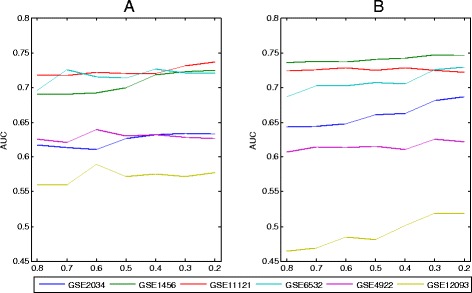
Effect of parameter *α* on the resulting AUC of RLR-EN and RLRNPI-EN models. The median AUC are obtained over 100 experiments based on 10-fold cross validation for *λ*. **a** RLR-EN. **b** RLRNPI-EN

There are three parameters *α*,*λ* and *r* in the proposed model which require multi-parameter optimization based on cross-validation and grid search. However, when sample sizes are small, the performances of the proposed model with different parameters are often same or similar. So it is difficult to choose a proper parameter combination. In addition, a grid search for three parameters takes a long time. Therefore, it is useful to fix all but one parameter and select a model based on that parameter. We investigate the effect of *α* only on methods RLR-EN and RLRNPI-EN, where we do not need to consider the value of *r*. We run the RLR-EN and RLRNPI-EN models on the six datasets with *α*=0.8,0.7,0.6,0.5,0.4,0.3,0.2. The median AUC based on 10-fold cross-validation for *λ* is shown in Fig. 2. For both RLR-EN and RLRNPI-EN models, the results indicate that the classification processes are very similar with a small perturbation of *α* when *α* falls to [0.2,0.4]. Thus, the value of *α* will be set to 0.3 for all the five models related with the elastic net.

**Fig. 3:**
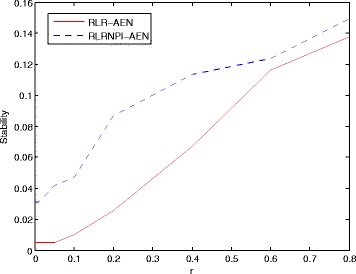
Effect of parameter *r* on the stability of RLR-AEN and RLRNPI-AEN models in between-dataset setting. The median stability of the top 100 genes are computed using all samples on each dataset. The genes are ranked by the number of times that the variables become selected when *λ* decreases. The results are computed by taking the median stabilities of the total pairs of all the six datasets

**Fig. 4:**
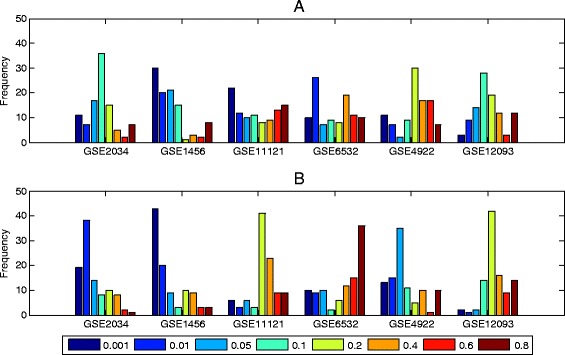
The selected frequency of each *r* in 100 experiments for RLR-AEN and RLRNPI-AEN models. The chosen (*λ*,*r*) is the one giving the highest AUC over 10-fold cross validation. If there are more than one combination (*λ*,*r*) that reach the highest AUC, we will choose the one that has the largest *r*. **a** RLR-AEN. **b** RLRNPI-AEN

The value of parameter *r* will result in a trade-off between stability of gene selection and accuracy of classification in the model with adaptive elastic net. We will consider the stability of RLR-AEN and RLRNPI-AEN in the between-dataset setting with different value of *r* (*r*∈{0.001,0.01,0.05,0.1,0.2,0.4,0.6,0.8}). According to the definition of weight in (8), the larger value of *r* will enhance the influence of the degrees of the variables on the models which do not change with different samples. Figure 3 compares the median stability of the top 100 genes estimated by RLR-AEN and RLRNPI-AEN with different values of *r* using all samples on each dataset. With the increase of *r*, there is a clear growth trend for the median stability over total pairs of all the six datasets. This phenomenon accords with the theoretical analysis. However, on the other hand, a larger *r* may result in a poor classification process. A two-parameters grid search based on 10-fold cross-validation on the training set is used to find the proper value of *r* for each dataset. The chosen (*λ*,*r*) is the one giving the largest AUC over 10-fold cross-validation which may be not the same under different random partition for training and testing sets. If there are more than one combination (*λ*,*r*) that reach the biggest AUC, we will choose the one that has the largest *r*. Figure 4 presents the selected frequency for each *r* in 100 (10 × 10-fold) experiments. In order to avoid a two-parameters grid search in the following experiments and the instability brought by different *r* selected based on different samples, we attempt to use a fixed *r* for each dataset, which is set to the most frequently occurring value in 100 experiments. According to Fig. 4, the value of *r* for each dataset is presented in Table 2. It is vary with the type of dataset for RLRNPI-AEN. The datasets considering the time for distant metastasis-free survival tend to choose the model with small *r*, while the other two types of datasets prefer the model with larger *r*.

**Table 2:** The value of *r* used in RLR-AEN and RLRNPI-AEN for each dataset

Dataset	GSE2034	GSE1456	GSE11121	GSE6532	GSE4922	GSE12093
RLR-AEN	0.1	0.001	0.001	0.01	0.2	0.1
RLRNPI-AEN	0.01	0.001	0.2	0.8	0.05	0.2

### Accuracy of the classification

Given the fixed *α* and *r*, we assess the predictive accuracy of the model based on a 10-fold cross-validation which is repeated ten times on each dataset. Table 3 shows the median AUC and the adjusted *p*-values for the seven models, where the *p*-values evaluate the significance of difference in classification performance between RLRNPI-AEN and the other six methods based on Mann-Whitney U test. For each dataset, the *p*-values are corrected using Holm-Bonferroni method for multiple testing. The Holm-Bonferroni method is more suitable for the multiple testing with small number of individual hypotheses and offers a simple test uniformly more powerful than the Bonferroni correction [47]. In general, classifiers using biological network information (RLR-AEN, RLRNPI-EN, ENCAPP, SVM-AEP and RLRNPI-AEN) have higher predictive power than those using gene expression dataset only (RLR-EN and limma). The results show that RLRNPI-AEN consistently outperforms limma with adjusted *p*-values <0.05 for all the six datasets except GSE4922. Since the value of *r* used in the adaptive elastic net for RLRNPI-AEN is not large for datasets GSE2034, GSE1456 and GSE4922, the classification performances of RLRNPI-EN and RLRNPI-AEN do not have significant difference. RLR-AEN, RLRNPI-EN and SVM-AEP have similar performances which are significantly different from those of RLRNPI-AEN for datasets GSE11121, GSE6532 and GSE12093. ENCAPP generally achieve the second best classification performance. RLRNPI-AEN has the manifest superiority in the case of small sample size, especially for datasets GSE11121 and GSE12093 (adjusted *p*-values <0.05 compared with all the other six methods). The network-based pairwise interaction may help the model away from the effects of noise and instability brought by the small sample size.

**Table 3:** Prediction performance of RLRNPI-AEN in comparison to other methods in terms of area under ROC curve (AUC)

Dataset	RLR-EN	RLR-AEN	RLRNPI-EN	limma	ENCAPP	SVM-AEP	RLRNPI-AEN
GSE2034	0.638	0.663	0.657	0.627	**0.681**	0.647	**0.690**
	**(0.0219)**	(0.0516)	(0.0516)	**(0.0036)**	(0.5966)	(0.0516)	(–)
GSE1456	0.724	**0.734**	0.711	0.619	0.722	0.717	**0.736**
	(0.9768)	(0.9920)	(0.5600)	**(0.0114)**	(0.9920)	(0.6024)	(–)
GSE11121	0.736	0.725	0.739	0.542	**0.750**	0.695	**0.820**
	**(0.0171)**	**(0.0076)**	**(0.0250)**	**(0.0012)**	**(0.0250)**	**(0.0050)**	(–)
GSE6532	0.721	0.725	0.725	0.643	**0.730**	0.715	**0.747**
	**(0.0451)**	**(0.0424)**	**(0.0422)**	**(0.0012)**	(0.4725)	**(0.0219)**	(–)
GSE4922	**0.620**	0.611	0.611	0.606	0.593	**0.622**	0.614
	(1.0000)	(1.0000)	(1.0000)	(1.0000)	**(0.1032)**	(1.0000)	(–)
GSE12093	0.571	0.518	0.613	**0.685**	0.616	0.607	**0.845**
	**(0.0012)**	**(0.0012)**	**(0.0012)**	**(0.0208)**	**(0.0034)**	**(0.0012)**	(–)

The median AUC obtained for each method on the six datasets over ten times ten-fold cross validation. The adjusted *p*-values calculated using a Mann-Whitney U test are shown within parentheses, which evaluate the significance of difference in classification performance between RLRNPI-AEN and the other six methods. For each dataset, they are corrected using Holm-Bonferroni method for multiple testing. The best two median AUCs and the adjusted *p*-values that are less than 0.05 are shown in boldface

### Stability of gene selection process

**Fig. 5:**
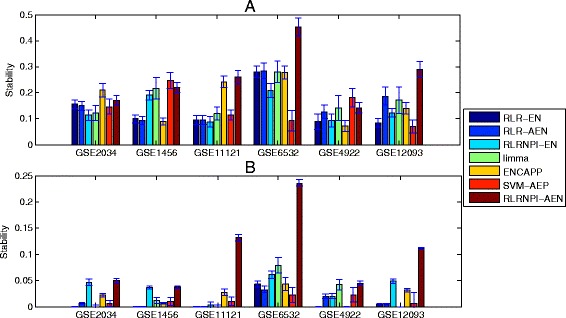
Stability for the selected genes based on 10-fold cross-validation. Median and standard errors are obtained over the 20 times random sampling of subsets. **a** Soft-perturbation setting. **b** Hard-perturbation setting

**Fig. 6:**
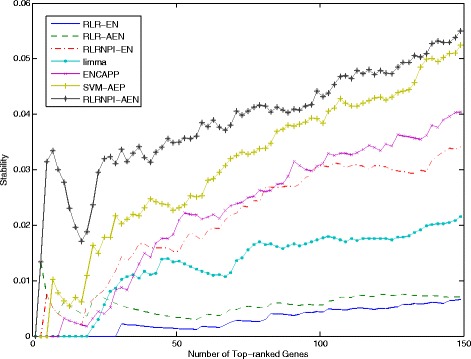
Stability for the top-ranked genes in between-datasets setting based on all samples on each dataset. The stabilities are obtained by taking the median of the pairwise stability measures. The x-axis is the number of selected genes

The stability of gene selection process is evaluated by Jaccard coefficient in three settings: the soft-perturbation, the hard-perturbation, and the between-datasets settings. Both soft-perturbation and hard-perturbation settings are based on the 10-fold cross-validation for the tuning parameter *λ*. Figure 5 shows the median stability of the selected genes estimated by seven models over 20 repeats of random sampling (The summary of median stability and adjusted *p*-values is presented in Additional file 2: Table S1). It appears very clearly that RLRNPI-AEN provides more stable gene selection process than the other six methods, especially in the hard-perturbation setting where the adjusted *p*-values are less than 10^−3^ in most cases. With a larger value of *r*, a significant stability is observable for datasets GSE11121, GSE6532 and GSE12093, using RLRNPI-AEN in soft-perturbation setting (adjusted *p*-values <0.05 except that of ENCAPP for GSE11121). This indicates that the idea of setting the weights for adaptive elastic net to be inversely proportional to the degrees is very useful for improving the stability of the gene selection. Since ENCAPP and SVM-AEP also take into account the biological network information, they have higher median stabilities in some of the datasets GSE2034, GSE1456 and GSE4922 in the soft-perturbation setting, where the value of *r* for RLRNPI-AEN is small. However, in these cases, the *p*-values indicate that the differences are not significant. In the between-datasets setting, another way is used to present the ability of one gene selection method in identifying the similar gene set for the same cancer from different datasets. We use all samples of each dataset. For the five elastic net based methods, given a decreasing sequence of values for *λ*, the genes are ranked by the number of times that they are selected. For limma and SVM-AEP, the genes are sorted in increasing order by the *p*-values of the corresponding tests, i.e., the moderated t-statistic test for limma and the test related with GO category’s enrichment analysis for SVM-AEP. Figure 6 gives the stabilities for the seven methods which are obtained by taking the median of pairwise stability measures. On one hand, for all the seven methods, the stability rise with the number of the top genes. The bigger gene sets will have more chance to share some genes. On the other hand, the four methods that consider biological network information yield much more stable biomarker selection than RLR-EN, RLR-AEN and limma. Because RLRNPI-AEN uses pairwise interactions based on both gene expression dataset and biological network to identify marker genes, it can retrieve significantly more overlapped biomarkers. For example, among the top-100 genes estimated by RLRNPI-AEN from datasets GSE11121 and GSE6532 which both consider the time for distant metastasis-free survival, there are 20 common genes, while RLR-EN, RLR-AEN, RLRNPI-EN, limma, ENCAPP and SVM-AEP only identify 2, 1, 8, 0, 5 and 2 common genes, respectively (The number of common genes between each pair of datasets among the top-100 genes is presented in Additional file 3: Table S2). Our results indicate that RLRNPI-AEN is an effective way to produce a more stability gene set.

### Functional stability

**Fig. 7:**
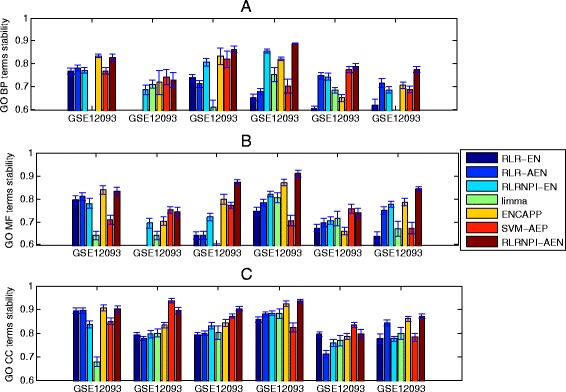
GO stability for the selected genes in soft-perturbation setting based on 10-fold cross-validation. Median and standard errors are obtained over the 20 times random sampling of subsets. **a** BP term. **b** MF term. **c** CC term

**Fig. 8:**
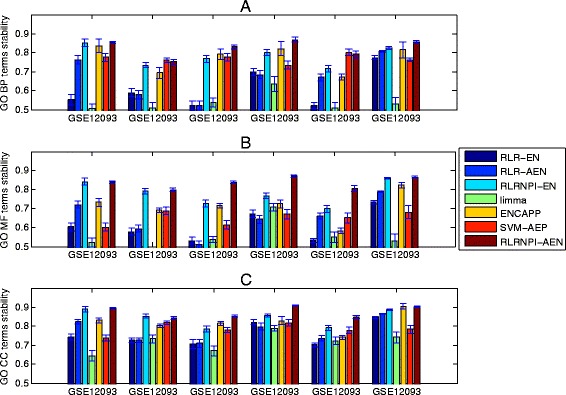
GO stability for the selected genes in hard-perturbation setting based on 10-fold cross validation. Median and standard errors are obtained over the 20 times random sampling of subsets. **a** BP term. **b** MF term. **c** CC term

**Fig. 9:**
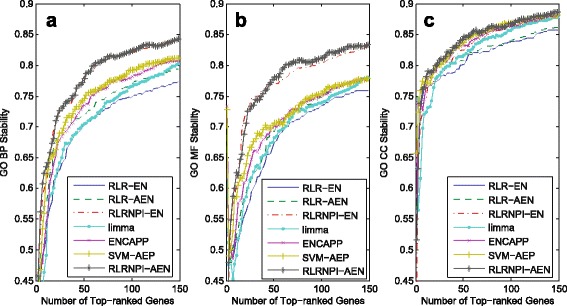
GO stability for the top-ranked genes in between-datasets setting based on all samples on each dataset. The GO stabilities are obtained by taking the median of the pairwise stability measures. The x-axis is the number of selected genes. **a** BP term. **b** MF term. **c** CC term

Since many gene selection methods are based on the samples, it is not easy to reach a high stability in term of genes with the change of samples. However, these biomarker sets with little common genes may exhibit the same biological function which also make sense for cancer diagnosis, treatment, and prognosis. Therefore, it is important for one gene selection method to identify some specific biological function related with the cancer in a robust manner. The stability analysis of the model in terms of biological function is implemented by using R package “GOSemSim” [42]. We assess on Figs. 7 and 8 the functional stability of all methods in the soft-perturbation and hard-perturbation settings, respectively. The three GO domains are analyzed separately (The summary of the median GO stability and adjusted *p*-values for the three GO domains BP, CC and MF in soft-perturbation and hard-perturbation settings is presented in Additional file 4: Table S3). From Figs. 7, 8 and Additional file 4: Table S3, we can find that the stability results at the functional level are very similar to the results at the gene level. Overall, RLRNPI-AEN is the most stable method. The advantages of the network-based methods in GO stability further show the strength of biological networks for achieving more clear biological interpretation. Since the sample difference is relatively small in soft-perturbation setting, the functional stabilities of the ENCAPP, SVM-AEP and RLRNPI-AEN models do not have large distinction for some datasets such as GES2034. However, RLRNPI-AEN exhibits significant functional stability benefits in hard-perturbation setting where there is no overlap in samples. In addition, the functional stability results with the change of the number of the top genes for the seven methods in between-datasets setting are presented in Fig. 9. In general, RLRNPI-EN and RLRNPI-AEN offers significant benefits in terms of stabilities for GO terms BP and MF. Another two methods ENCAPP and SVM-AEP which are integrated with biological network also perform better than RLR-EN, RLR-AEN and limma. There is no obvious difference between the functional stability of RLRNPI-EN and RLRNPI-AEN in this between-datasets setting. Since this pair of methods is based on similar ideas, they tend to identify genes with consistent functions. However, there are clear strengths of highlighting the heterogeneous underlying interactions.

### Biomarker identification

**Fig. 10:**
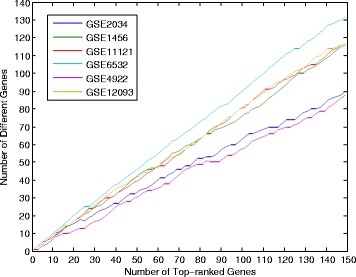
The number of the different genes among the top-k genes selected by RLR-AEN and RLRNPI-AEN. The x-axis is the number of selected marker genes ranked by the number of times that the variables become selected when regularization parameter decreases. The y-axis is the number of the different genes that do not selected by RLR-AEN and RLRNPI-AEN simultaneously

The other five methods focus on the differentially expressed genes or modules, while RLRNPI-EN and RLRNPI-AEN identify the gene interactions of which the changing may result in different states of a biological system. Figure 10 presents the number of the different genes among the top-k genes selected by RLR-AEN and RLRNPI-AEN, using all the samples on each dataset. Although these numbers are vary with different datasets, all the results indicate that there are obvious difference between the biomarkers identified by RLR-AEN and RLRNPI-AEN. We will make a detailed analysis of the biomarkers for datasets GSE1456 and GSE11121.

**Fig. 11:**
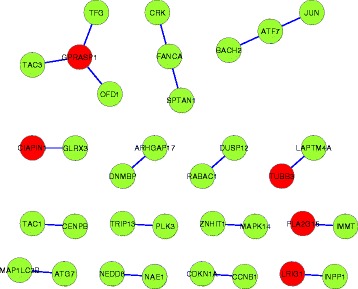
The subnetworks for dataset GSE1456 estimated by RLRNPI-AEN. Nodes represent human genes, and they are connected by a link if they belong to the PPI network. Each gene is labeled by its gene symbol. The color of each node indicates whether the gene are identified by RLR-AEN (*red*) or not (*green*)

For GSE1456, we first decide the number of biomarkers based on AUC by 10-fold cross-validation on the whole dataset. To reduce the variability, the cross-validation is conducted 10 times. Then we select the top-k variables which are sorted by the number of times that the variables become selected when *λ* decreases, where *k* is set to the median biomarker number over the 10 repeated experiments. The subnetworks based on the 19 interactions identified by RLRNPI-AEN are presented in Fig. 11, where the color of each node indicates whether the gene belong to the top-19 genes estimated by RLR-AEN (red) or not (green). There are 34 genes selected as informative, including 5 expression-based discriminative genes that belong to the 19 genes selected by RLR-AEN (The 19 important interactions identified by RLRNPI-AEN for datasets GSE1456 are presented in Additional file 5: Table S4). RLR-AEN tends to select a part of genes of the subnetwork which are differentially expressed and neglect the genes who interact with some discriminative genes to form a collective biological function or present differential correlation under many kinds of biological states. The functional and biological relationships of the selected genes of each subnetwork are analyzed based on the GO annotation, which is implemented by using R package “clusterProfiler” [48]. A *p*-value of a GO terms set transferred from a gene set is calculated using the hypergeometric distribution and then is adjusted using the FDR correction for multiple testing. Table 4 lists the GO terms with the smallest adjusted *p*-value for some subnetworks shown in Fig. 11 at 5 % FDR. The small *p*-value shows that the genes in each subnetwork have significant biological and functional correlation, and the common GO functions they share are often related to the relapse time of breast cancer.

**Table 4:** The Gene Ontology results of the subnetwork identified by RLRNPI-AEN for dataset GSE1456

Subnetwork	GO number	Ontology description	Adjusted *p*-value
TFG GPRASP1 TAC3 OFD1	GO:0043015	gamma-tubulin binding	3.07×10^−2^
	GO:0043014	alpha-tubulin binding	3.07×10^−2^
SPTAN1 CRK FANCA	GO:0045309	protein phosphorylated amino acid binding	3.02×10^−2^
	GO:0030507	spectrin binding	3.02×10^−2^
	GO:0046875	ephrin receptor binding	3.02×10^−2^
	GO:0042169	SH2 domain binding	3.02×10^−2^
	GO:0051219	phosphoprotein binding	3.48×10^−2^
BACH2 ATF7 JUN	GO:0000977	RNA polymerase II regulatory region sequence-specific DNA binding	2.61×10^−4^
	GO:0001012	RNA polymerase II regulatory region DNA binding	2.61×10^−4^
	GO:0000976	transcription regulatory region sequence-specific DNA binding	2.61×10^−4^
	GO:0000981	sequence-specific DNA binding RNA polymerase II transcription factor activity	2.61×10^−4^
	GO:0000980	RNA polymerase II distal enhancer sequence-specific DNA binding	2.61×10^−4^

The first column (Subnetwork) presents the elements of subnetwork of which the functional and biological relationship are analyzed based on the GO annotation

Specifically, the smallest adjusted *p*-value is 2.61×10^−4^ corresponding to GO:0000977 which is related to RNA polymerase II regulatory region sequence-specific DNA binding. All the genes in the third subnetwork share this common GO function, which are not identified by RLR-AEN. It is a molecular function related with RNA polymerase II which plays an important role in breast [49, 50].

**Fig. 12:**
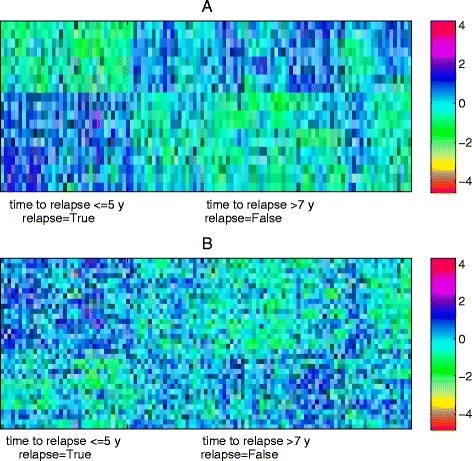
The heatmap of the expression profile of the 19 interactions identified by RLRNPI-AEN and their corresponding genes for dataset GSE1456. **a** Interactions (**b**) Genes

Figure 12 shows the heatmap of the expression profile of the involved 34 genes and the 19 interactions. The distinction between two classes are more significant in edge data than that in the gene expression, which shows that our proposed method does select reasonable biomarkers for classification. Most of identified biomarkers are considered to have diagnostic values for breast. For example, the suppression of LRIG1 gene of the top-1 edge is identified as a common feature of breast tumors, and contributes to poor patient prognosis and therapeutic resistance [51]. TAC1 has been implicated in the development of breast which can lead to the production of cytokines with growth promoting functions [52]. In addition, CIAPIN1 is reported to participate in breast cancer multi-drug resistance, changing cell cycle and enhancing the anti-apoptotic capability of cells [53].

Unlike conventional regularized logistic regression, our model with network-based pairwise interaction can implicate disease-related genes with low discriminative potential, such as ATF2 and OFD1. The activation of ATF2 has been detected to play a direct role in malignant phenotypic changes of human breast epithelial cells [54]. Furthermore, OFD1 is reported to be possible to reverse the cilia-defective phenotype of a transformed breast cancer cell line [55].

**Fig. 13:**
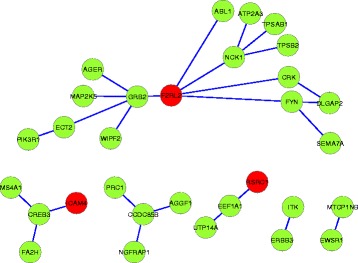
The subnetworks for dataset GSE11121 estimated by RLRNPI-AEN. Nodes represent human genes, and they are connected by a link if they belong to the PPI network. Each gene is labeled by its gene symbol. The color of each node indicates whether the gene are identified by RLR-AEN (*red*) or not (*green*)

Next, the top-26 interactions for dataset GSE11121 are also analyzed, including 31 informative genes of which 3 are shared with RLR-AEN (The 26 important interactions identified by RLRNPI-AEN for datasets GSE11121 are presented in Additional file 5: Table S4). Figure 13 presents the network of the top 26 interactions. Different from the subnetworks with a few nodes for GSE1456, the biomarkers tend to form a larger subnetwork for GSE11121. The larger value of *r* for GSE11121 may make RLRNPI-AEN select the genes with higher degree. It indicates that there may exist different modes between breast metastasis and relapse. The GO results for some subnetworks are shown in Table 5. It can be seen from this table that genes in each subnetwork share some common GO functions with high statistical significance, indicating high biological and functional correlation of the genes in this subnetwork. Therefore, the genes without differentially expressed remain essential for maintaining the integrity of the subnetwork. For the first subnetwork, GO:0002433 with adjusted*p*-value 2.66×10^−10^ is a biological process shared by seven genes among 16 genes, which is related to immune response-regulating cell surface receptor signaling pathway involved in phagocytosis. There are results demonstrate that phagocytosis of extracellular matrix is an inherent feature of breast tumor cells that correlates with and may even directly contribute to their invasive capacity [56].

**Table 5:** The Gene Ontology results of the subnetwork identified by RLRNPI-AEN for dataset GSE11121

Subnetwork	GO number	Ontology description	Adjusted *p*-value
AGER ATP2A3 CRK CSF2RA DLGAP2 ECT2 F2RL2 FYN GRB2 MAP2K5 NCK1 PIK3R1 SEMA7A TPSAB1 TPSB2 WIPF2	GO:0002433	immune response-regulating cell surface receptor signaling pathway involved in phagocytosis	2.66×10^−10^
	GO:0038096	Fc-gamma receptor signaling pathway involved in phagocytosis	2.66×10^−10^
	GO:0038094	Fc-gamma receptor signaling pathway	2.66×10^−10^
	GO:0002431	Fc receptor mediated stimulatory signaling pathway	2.70×10^−10^
	GO:0006909	phagocytosis	5.53×10^−8^
CREB3 MS4A1 ICAM4 FA2H	GO:0031726	CCR1 chemokine receptor binding	2.25×10^−2^
	GO:0008140	cAMP response element binding protein binding	2.25×10^−2^
	GO:0044877	macromolecular complex binding	2.25×10^−2^
	GO:0035497	cAMP response element binding	2.76×10^−2^
	GO:0005102	receptor binding	2.76×10^−2^
AGGF1 CCDC85B PRC1 NGFRAP1	GO:0005123	death receptor binding	4.17×10^−2^
	GO:0008656	cysteine-type endopeptidase activator activity involved in apoptotic process	4.17×10^−2^
	GO:0016505	peptidase activator activity involved in apoptotic process	4.17×10^−2^
	GO:0019894	kinesin binding	4.17×10^−2^
	GO:0016504	peptidase activator activity	4.17×10^−2^

The first column (Subnetwork) presents the elements of subnetwork of which the functional and biological relationship are analyzed based on the GO annotation

**Fig. 14:**
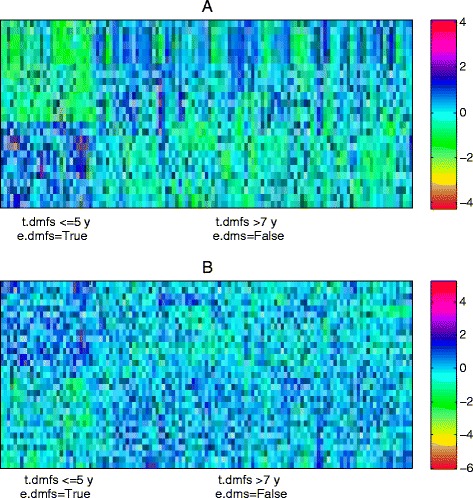
The heatmap of the expression profile of the 26 interactions identified by RLRNPI-AEN and their corresponding genes for dataset GSE11121. **a** Interactions (**b**) Genes

The heatmap of the interactions and the corresponding genes are presented in Fig. 14. It is consistent with the results presented in Fig. 12 that the opposite patterns between the two classes are clear in interaction data but not in the gene expression data. Some of the genes are well known in the literatures. Although one node of top-1 edge ICAM4 is not yet indicated to play an important role in breast cancer susceptibility, its role in cell adhesion and cell signaling together with its low level expression in cancer-relevant tissues leave the possibility that its dysregulation or dysfunction may increase cancer risk [57]. The other node CREB3 is not selected by RLR-AEN of which the over-expression is shown to substantially increase the migration of MDA-MB-231 metastatic breast cancer cells [58]. FYN which belongs to the top-2 edge is implicated in diverse biological functions such as neuronal development, T-cell receptor signaling, and is reported to be linked to increased breast cancer risk, especially in women with expression of ER and PR in their breast tumors [59]. GRB2 of the top-3 edge plays an important role in the first subnetwork. Over expression of GRB2 might modulate the growth factor sensitivity of human breast cancer cells and has influence on tumor progression [60].

## Conclusions

In this paper, we present an effective biomarker discovery and cancer classification algorithm: a regularized logistic regression with network-based pairwise interaction via adaptive elastic net. Different from the algorithm based on functional modules proposed by Das et. al in [3], where the modules often need to be determined in advance by some clustering methods and thus the algorithm performance may depend on the module detection, we focus on the gene pairs which exhibit different positive or negative interactions. The discriminative modules treat the genes and the interactions as a whole and do not consider the diversity of the interactions between a variety of diseases. Since the mutation of buried residues of the proteins leads to loss or gain of specific interactions, the interactions identified by the proposed method can be great helpful for the analysis of exposed residues in turn of which the mutation has been shown to be a higher fraction of mutations associated with autosomal-dominant diseases [25, 61]. In addition, by considering the changes of interactions towards different biological states, we can identify the non-differentially expressed genes which play central roles in functional process within cells. Our algorithm combines gene expression profiles with PPI networks, which can reduce the influence of noise brought from the correlation between expression that in fact have no underlying biological causality. The degree information based on the PPI network is introduced to make the model less sensitive to the training samples and predict biomarkers with higher reproducibility.

Since we only take account of the interactions between two genes, it may miss some informative biomarkers which do not participate in any interactions. Therefore, in the future work, the subnetworks or the pathways consisted of both nodes and edges are needed for the accuracy of diagnostic and prognostic biomarker identification. Although the network information introduced in our model facilitate the discovery of more reproductivity biomarkers, the results may be dependent on the network structure. With the availability of a variety of biological networks such as KEGG pathways, we can incorporate all these sources as prior information to build variable selection methods to decline the sensitive of the model towards both gene expression data and networks.

## Availability of data and materials

The datasets supporting the results of this article are included within its additional files.

## Additional files

Additional file 1
**The introductions for RLR-EN and RLR-AEN.** This section provides the brief introductions for the two compared methods: RLR-EN and RLR-AEN. (PDF 128 kb)

Additional file 2
**Table S1.** Summary of the median stability and the adjusted *p*-values for the different datasets in soft-perturbation and hard-perturbation settings. (XLSX 12.9 kb)

Additional file 3
**Table S2.** Summary of the number of common genes between each pair of datasets among the top-100 genes. (XLSX 10.4 kb)

Additional file 4
**Table S3.** Summary of the median GO stability and the adjusted *p*-values for the three GO domains (BP, CC, MF) in soft-perturbation and hard-perturbation settings. (XLSX 18.7 kb)

Additional file 5
**Table S4.** The important interactions identified by RLRNPI-AEN for datasets GSE1456 and GSE11121. (XLSX 10.3 kb)
