# Supplementary material for: Regularized logistic regression with network-based pairwise interaction for biomarker identification in breast cancer
Source: BMC Bioinformatics. 2016 Feb 27;17:108. doi: 10.1186/s12859-016-0951-7 (PMC4769543; doi:10.1186/s12859-016-0951-7)
Supplement: Additional file 1 — The introductions for RLR-EN and RLR-AEN. This section provides the brief introductions for the two compared methods: RLR-EN and RLR-AEN. (PDF 128 kb) [file 12859_2016_951_MOESM1_ESM.pdf]

Regularized logistic regression with network-based pairwise interaction for biomarker identification in breast cancer: Additional file

Meng-yun Wu, Xiao-Fei Zhang, Dao-Qing Dai, Le Ou-Yang, Yuan Zhu and Hong Yan

1 The regularized logistic regression via adaptive elastic net

Suppose that there are n independent p-dimensional observations, with binary response vector $y = (y_1, \cdots, y_n)^T$ and design matrix $X = (x_1, \cdots, x_n)^T$, where $x_i = (x_{i1}, x_{i2}, ..., x_{ip})$ and $y_i \in \{0, 1\}$.

The regularized logistic regression maximize the penalized log-likelihood

$$\frac{1}{n}\sum_{i=1}^{n}\left[y_i \log p^{(2)}(x_i) + (1-y_i)\log(1-p^{(2)}(x_i))\right] - \lambda P_{\alpha}^{(2)}(\beta), \tag{1}$$

where $p^{(2)}(x_i)$ is represented by a linear function of the variables as follows

$$p^{(2)}(x_i) = Pr(y_i = 1|x_i) = \frac{1}{1+e^{-(\beta_0 + x_i^T\beta)}}, \tag{2}$$

and $P_{\alpha}^{(2)}(\beta)$ is a penalty function which can shrink some components of β to zero for some appropriately chosen λ and α [1]. The log-likelihood part of (1) can be written as

$$\frac{1}{n}\sum_{i=1}^{n}\left[y_i\left(\beta_0 + \sum_{j=1}^{p}\beta_j x_{ij}\right) - \log\left(1 + e^{\beta_0 + \sum_{j=1}^{p}\beta_j x_{ij}}\right)\right], \tag{3}$$

The regularized logistic regression via adaptive elastic net (RLR-AEN) considers the above model based on the adaptive elastic net penalty

$$P_{\alpha}^{(2)}(\beta) = \sum_{j=1}^{p}\left[\frac{1}{2}(1-\alpha)\beta_j^2 + \alpha w_j^{(2)}|\beta_j|\right], \tag{4}$$

which is a compromise between the ridge-regression penalty ($\alpha = 0$) and the adaptive lasso penalty ($\alpha = 1$) [2]. The weights for adaptive elastic net (4) in objective function (1) are set to be inversely proportional to the degrees as follows,

$$w_j^{(2)} = (d_j)^{-r} \tag{5}$$

where d_j is the degree of node j in the biological network. When $r = 0$, the model turns back to the regularized logistic regression via elastic net (RLR-EN). The estimators of both methods RLR-EN and RLR-AEN can be obtained by matlab code “glmnet”.

References

[1] Jerome Friedman, Trevor Hastie, and Rob Tibshirani. Regularization paths for generalized linear models via coordinate descent. *Journal of Statistical Software*, 33(1):1–22, 2010.

[2] Hui Zou and Hao Helen Zhang. On the adaptive elastic-net with a diverging number of parameters. *Annals of Statistics*, 37(4):1733–1751, 2009.
